# 

**Published:** 1862-11

**Authors:** 


### CONTENTS.

### ORIGINAL CONTRIBUTIONS.

ART. XXXV. LIGATURE OF THE CAROTID ARTERY. By H. WARDNER, M.D., Brigade-Surgeon, .......... 651

ART. XXXVI. CASES REPORTED FOR THE MEDICAL EXAMINER. By O. B. ORMSBY, M.D., Assistant-Surgeon, 18th Ill., .......... 654

ART. XXXVII. THE MEDICINAL USES OF ERGOT, OTHER THAN AS A PARTURIFACIENT. By M. O. HEYDOCK, M.D., .......... 658

ART. XXXVIII. ON THE TREATMENT OF CANCEROUS DISEASES. By E. MARGUERAT, M.D., of Chicago, .......... 662

### ARMY CORRESPONDENCE.

LETTER FROM E. ANDREWS, M.D., .......... 669

### SELECTIONS.

Experimental Researches into a new Excretory Function of the Liver; Consisting in the Removal of Cholesterine from the Blood, and its Discharge from the Body in the form of Stercorine. (*The Seroline of Boudet.*) By Austin Flint, Jr., M.D., Professor of Physiology and Microscopy in the Bellevue Medical College, New York; &c... 671

Salivary Calculus, .......... 697

Vaccination in Whooping-Cough, .......... 698

### BOOK NOTICES.

The Dentists's Memorandum. A Book of Engagements, and Manual of Ready Reference, for 1863. By C. M. Cleveland, M.D., Cincinnati: Bradley & Webb, Printers, .......... 699

On Medical Provision for Railroads, as a Humanitarian Measure, as well as a Source of Economy to the Companies. By Edmund S. F. Arnold, M.D., Fellow of the New York Academy of Medicine, &c........ 699

Charter, Constitution, By-Laws, and Fee-Bill of the Eaton Medical Society, at Eaton, Ohio, .......... 700

### EDITORIAL.

Wm. Hopp's on Trial for Murder. A Medico-Legal Case, .......... 700

American Medical Association, .......... 705

Transactions of the New York Academy of Medicine, .......... 706

Sick and Wounded in Washington and Vicinity, .......... 706

Surgeon J. V. Z. Blaney, United States Volunteers, .......... 706

Assistant-Surgeon C. C. Dumreicher, U.S.A., .......... 706

Provision for taking Care of the Wounded in Battle, .......... 706

The Surgeon-General's New Bill of Fare for the Hospitals, .......... 711

